# Suicidal Thoughts and Behaviors in People on the Autism Spectrum

**DOI:** 10.1007/s11920-024-01533-0

**Published:** 2024-09-30

**Authors:** Morganne Reid, Daylin Delgado, Julia Heinly, Bridgett Kiernan, Samantha Shapiro, Lisa Morgan, Brenna Maddox, Shari Jager-Hyman

**Affiliations:** 1 Department of Pediatrics, Emory University School of Medicine, Atlanta, GA USA; 2 Department of Psychiatry, TEACCH Autism Program, University of North Carolina - Chapel Hill, Carrboro, NC USA; 3 Department of Psychiatry, Perelman School of Medicine, University of Pennsylvania, 3535 Market Street, 3rd floor, Philadelphia, PA 19104 USA; 4 Lisa Morgan Consulting LLC, Kittery, ME USA; 5 Penn Leonard Davis Institute of Health Economics, University of Pennsylvania, Philadelphia, PA USA

**Keywords:** Autism, Suicidal ideation, Suicidal behavior, Suicide

## Abstract

**Purpose of Review:**

This review synthesizes recent research on suicidal thoughts and behaviors among autistic individuals. We present literature on risk and protective factors, risk assessment, intervention, and crisis services, and recommendations for future research.

**Recent Findings:**

Literature on this topic has grown substantially in recent years. Areas of advancement include improved understanding of risk factors (e.g., Interpersonal Theory of Suicide constructs, autistic burnout, mental health conditions, cognitive factors, diagnosis timing, emotion dysregulation), screening, assessment, acute-care services, and suicide-specific psychosocial treatments (e.g., safety planning, dialectical behavior therapy). Gaps include protective factors, impact of intersectional identities, and tailored approaches to screening, assessment, and intervention.

**Summary:**

Heightened awareness of suicide risk in autistic individuals has led to increased understanding of why autistic individuals think about and attempt suicide and the strategies used to identify and reduce suicide risk. We recommend community-partnered, multi-faceted, and strength-based approaches to inform tailored prevention and intervention efforts.

## Introduction

Suicide is a leading cause of death and a significant contributor to premature mortality among autistic individuals^1^ [1]. A growing body of research suggests that rates of suicidal thoughts and behaviors (STB) are higher among autistic individuals than non-autistic individuals [2–4]. The autistic community and autism researchers have identified STB as a priority area of focus [5–7]. Synthesizing the recent literature on this topic can provide a framework to guide future suicide prevention efforts for autistic people.

There are several published systematic reviews on STB in autistic people. While the first review published in 2013 included only four studies of autistic people aged 25 or younger [8], the two most recent reviews included 45 articles focused on adults [9] and 47 studies on youth [10] reflecting increased attention to this important issue. In the present article, we review the recent literature (i.e., from 2021 to 2022) focused on STB among autistic individuals across the lifespan, with the goals of highlighting new findings and identifying key gaps in the research. We first review literature on the Interpersonal Psychological Theory of Suicide (IPTS) as a potential framework for STB risk factors, followed by recent studies on other risk and protective factors for STB in autistic people. Next, we present literature on suicide risk screening and assessment, crisis services, and treatment for STB in the autistic population. Finally, we provide recommendations for future directions.

## Interpersonal Psychological Theory of Suicide (IPTS)

A critical step for reducing STB in the autistic community is understanding why many autistic people think about suicide and why a subset of these individuals attempt suicide. Several existing theories identify factors, both distal and proximal, that confer risk for STB in the general population. One theory with extensive empirical support in non-autistic people, Joiner’s [11].

IPTS, may also provide a useful framework for understanding STB in autistic individuals (e.g [12]). Briefly, IPTS posits that two factors are necessary for a suicide attempt to occur: an individual must desire to die and must be capable of acting on this desire. According to IPTS, the desire to die is fueled by a combination of *thwarted belongingness* -- an individual’s need to belong is unmet -- and *perceived burdensomeness* -- an individual’s perception that others are burdened and would be better off without them. IPTS also postulates that the capability for suicide is acquired after repeated exposure to painful experiences (e.g., non-suicidal self-injury, non-lethal suicide attempts, abuse), resulting in elevated tolerance to pain and reduced fear of death over time (hereafter referred to as *acquired capability)*. At face value, IPTS seems relevant for autistic people given high rates of experiences that may confer vulnerability to each key construct. Recent research has begun to examine the various components of IPTS and their contributions to STB in autistic individuals.

### Thwarted Belongingness, Perceived Burdensomeness, and Related Experiences

Despite a common assumption that autistic people are not motivated by social factors, it is now clear that social connection and/or a sense of belonging are critical to well-being for many autistic individuals [9, 13]. Unfortunately, many autistic individuals endure a host of experiences that negatively impact their sense of belongingness, including social dissatisfaction, loneliness, and invalidation [14, 15]. Similarly, experiences associated with perceived burdensomeness (e.g., unemployment, homelessness) are also relatively common among autistic people (e.g [16–18]).

To “fit in” in a world designed for neurotypical individuals and avoid the negative experiences described above, autistic people may *camouflage* or *mask* their autistic traits. Building on previous findings [19, 20], recent research highlights potential psychological costs of camouflaging. For example, Bradley et al. [21] queried 277 adults who self-reported an autism diagnosis or self-identified as autistic, about their experiences with camouflaging and identified dangers of camouflaging, including STB, as a common theme. They also found that the amount of time spent camouflaging is critical, with more time associated with worse psychological outcomes.﻿

Like camouflaging, *loneliness* is a state adjacent to thwarted belongingness commonly reported by autistic individuals that could contribute to STB [22]. In a study of 166 autistic youth with co-occurring anxiety disorders and/or obsessive-compulsive disorder, La Buissonnière-Ariza et al. [23] found a positive association between perceived loneliness and suicidal ideation (SI), even when controlling for age, sex, and IQ.

### Acquired Capability for Suicide

Autistic individuals report high rates of lifetime exposure to painful experiences commonly associated with acquired capability, including childhood abuse, non-suicidal self-injury, bullying, victimization, and maltreatment [24–26]. Liu et al. [26] demonstrated that experiencing more than one painful, fearful, or traumatic experience may be particularly pernicious with regards to suicide risk. In their sample of 219 autistic adolescent-parent dyads, adolescents exposed to multiple types of harassment (e.g., social exclusion, verbal bullying, beaten up, stolen items) were more likely to report SI or suicide attempts than those who reported a single type of harassment [26].

In alignment with the IPTS, Moseley et al. [12] found that, among 314 autistic adult survey respondents, reduced fear of death (but not pain tolerance) was indeed associated with a greater number of lifetime suicide attempts; however, this association was not found for increased pain tolerance. The authors noted that their null pain-tolerance finding should be interpreted with caution given limitations of the 2-item subscale they used to assess this construct. Moseley et al. [12] also found that in autistic individuals, non-suicidal self-injury had indirect effects on more lifetime suicide attempts, and this relationship was mediated by mental rehearsal and reduced fear of death. More research is needed to inform an expanded conceptualization of acquired capability that includes mental rehearsal of suicide plans. One facet to explore is whether mental rehearsal is more detailed and realistic for autistic people given a common autistic strength of visual thinking [27].

Although there is now evidence of the relevance of at least some components of IPTS for autistic individuals, future research should also consider the application of other major STB theories. One potentially applicable theory is Klonsky’s action-to-ideation Three-Step Theory, which espouses that suicidal behavior occurs when the following conditions are met: (1) concurrent pain **and** hopelessness, which leads to the desire for suicide; (2) pain exceeds or overwhelms connectedness; and (3) acquired capability [28]. It is also important to investigate novel frameworks specific to autistic individuals, given their unique risk and protective factors, as discussed in more detail below.

## Additional Proximal and Distal Risk Factors

### Autistic Burnout

Autistic burnout refers to a debilitating experience characterized by exhaustion, withdrawal, diminished executive functioning, reduced tolerance to stimuli, and loss of skills due to the stress of living in a neurotypical world [29, 30]. Autistic burnout can be the result of the stress and energy it takes to *camouflage* or *mask* one’s autistic characteristics in an unaccommodating world and is distinct from occupational burnout or clinical depression [29, 30]. The cycle of camouflaging and experiencing thwarted belongingness, despite efforts to mask autistic traits [14], leaves autistic individuals burnt out, exhausted, and hopeless overtime [29]. Although quantitative examinations have yet to be published, qualitative findings support an association between autistic burnout and STB (e.g [29–31]). Mantzalas et al. [31] completed a thematic analysis of 1,127 public online platform posts about autistic burnout and identified an association between autistic burnout and STB, particularly in the context of co-occurring mental health conditions. When experiencing autistic burnout, autistic individuals may be more vulnerable to STBs and have a reduced ability to engage in coping techniques [31]. Importantly, research suggests that camouflaging, a contributor to autistic burnout, significantly predicts STBs after controlling for other factors, such as age, sex, developmental condition (e.g., learning disability, Attention-Deficit/Hyperactivity Disorder), depression, anxiety, employment, and satisfaction with living arrangements [24]. This suggests that factors that contribute to autistic burnout, such as camouflaging, may be independent risk factors for STB.

### Co-occurring Mental Health Conditions

Researchers have identified links between STB and mental health symptoms and conditions in autistic individuals (e.g [3, 32]). In a nationwide retrospective cohort study from Denmark, co-occurring psychiatric conditions (e.g., depression, anxiety, schizophrenia) were found to confer risk for suicide attempts and deaths in autistic individuals aged 10 years or older [3]. In addition, Ellison et al. [32] found that elevated levels of parent-reported affective and externalizing problems were significantly associated with SI in autistic youth.

Some studies suggest that co-occurring psychiatric disorders may independently explain heightened suicide risk among autistic individuals. For example, in a large birth cohort, Jokiranta-Olkoniemi et al. [33] found that elevated suicide risk among autistic individuals relative to non-autistic individuals was no longer significant when controlling for co-occurring psychiatric conditions. In contrast, other studies have found that autistic traits independently predict SI, after controlling for depression [34, 35]. Thus, more research is needed to better understand the relationship between co-occurring mental health conditions and STB, as well as the underlying mechanisms.

### Cognitive Factors

Understanding the relationship between cognitive functioning and STB can inform identification of suicide risk in the autistic population. Casten et al. [36] examined associations between cognitive ability, SI, and autism across three groups: one from a clinic specializing in autistic youth with exceptional cognitive ability (i.e., estimated IQ at or above the 90th percentile), one from a large national autism study, and a general population cohort. Across these samples, they found higher rates of SI in autistic individuals relative to IQ-matched non-autistic individuals, and that autistic youth with SI had significantly higher cognitive ability than autistic youth without SI. Similarly, Gilmore et al. [37] found that autistic older adults (65 + years) without intellectual disability (ID) were more likely to experience SI or intentional self-injury compared to autistic older adults with ID. Notably, Cervantes, Brown, and Horowitz [38] found that emergency department (ED) visits related to SI or intentional self-inflicted injury were more likely for autistic youth with co-occurring mild ID than autistic youth with severe ID, unspecified ID, or no co-occurring ID. Therefore, the relationship between STB and intellectual functioning in autistic people is complex and warrants additional investigation.

### Timing of Diagnosis

Late diagnosis of autism is another potential risk factor for STB [2]. Commensurate with this literature, Hosozawa et al. [39] followed a U.K. birth cohort (*n* = 11,320) from age 5 to age 14 and found that older age of autism diagnosis was associated with greater risk of intentional self-injury with or without suicidal intent. The authors suggest several underlying protective mechanisms, including the association between early diagnosis and early intervention and improved support at school, which may improve the child’s social and coping skills and protect against mental health problems in adolescence. On the other hand, the authors suggest that increased exposure to bullying-victimization resulting from later diagnosis could be one of the underlying mechanisms for greater risk of intentional self-injury [39]. In contrast, Moseley and colleagues [12] did not find an association between age at diagnosis and SI, attempts, or any IPTS risk factor in their online study with 314 autistic adults. However, the authors noted a lack of variance in age at diagnosis in their sample, which may explain the null finding.

### Emotion Dysregulation

Emotion dysregulation is a multifaceted construct comprising difficulties modulating the intensity and duration of emotional responses in accordance with an individual’s goals and situational demands [40]. Often characterized by anger reactivity and dysphoria, emotion dysregulation difficulties are more prevalent among autistic individuals than the general population. For example, Conner and colleagues [41] found that autistic 6- to 17-year-olds from community (*n* = 1,169) and psychiatric inpatient (*n* = 567) settings were respectively four and seven times more likely to exhibit emotion regulation impairment than a nationally representative non-autistic youth sample (*n* = 1,000). Although a robust literature has linked emotion dysregulation and STB in the general population association, little research has focused on this association in the autistic community.

### Protective Factors

Although STB protective and risk factors have both been identified as priorities for the autistic community and those who support them, the literature on protective factors remains underdeveloped [5]. Protective factors against suicide that have been identified for the neurotypical population do not consistently demonstrate the same strength or association when examined in the autistic population. For example, higher IQ and higher education may buffer against STB in the general population [42, 43] but not in the autistic population (e.g [25, 36]). Additionally, being married or cohabiting and employed are less protective in autistic than non-autistic individuals [3].

Despite the dearth of literature in this area, there are initial efforts to identify protective factors in this group. For example, recent literature reports that dog ownership may be protective against STB for autistic individuals [44]. Barcelos et al. [44] interviewed 36 autistic dog owners and found that 16.7% cited needing to care for their dog and receiving their dog’s affection as significant reasons to prevent them from taking their lives. In fact, participants linked the routine and responsibilities of being a dog owner as well as positive interactions with their dog to their purpose in life [44]. Despite these initial efforts, significantly more research into protective factors for autistic individuals is needed. One potential protective factor that warrants more investigation is validation of experience, which has been identified as a protective factor in mental health promotion for autistic individuals [6].

### Suicide Risk Screening and Assessment

Mental health clinicians feel significantly less confident screening autistic individuals for suicide risk compared to their neurotypical peers [45], and key suicide risk assessment questions designed for the general population may be interpreted differently by autistic individuals [46] or display poor validity and reliability with autistic youth [47].

Rybczynski et al. [48] used the Ask-Suicide Screening Questions (ASQ) tool [49] to examine the feasibility of routine screening, identification, and referral for suicide risk in pediatric neurodevelopmental disabilities medical clinics. While universal screening with the ASQ was feasible, it was unclear to what degree autistic individuals had difficulty understanding the questions, and the tool is not yet validated with autistic youth [48]. As such, researchers are modifying or developing suicide risk screening and assessment tools specifically for the autistic population. For example, there is an ongoing study examining the efficacy of the ASQ tailored for youth with neurodevelopmental disorders [50]. Cassidy et al. [51] used feedback from autistic adults to adapt the Suicidal Behaviours Questionnaire-Revised (SBQ-R) and validated the resulting SBQ-Autism Spectrum Conditions (SBQ-ASC) to identify STB in autistic and possibly autistic adults participating in research. Additionally, Hedley et al. [2] found that the Suicidal Ideation Attributes Scale (SIDAS) [53], modified with autistic individuals’ input (SIDAS-M), may be appropriate for identifying SI in autistic adults without ID.

Some modifications included adding visual aids (e.g., thermometer, visual analogue scale), ratings to capture “sticky” suicidal thoughts (e.g., thoughts which fall between “brief passing thought” and “planned suicide”), and qualitative questions. Edits to the language were made to clarify terms (e.g., define suicide attempt; distinguish between non-suicidal and suicidal self-harm), be more concrete and direct, and eliminate implication of moral judgment (e.g., “commit suicide”) [51, 52]. Ongoing efforts in screening and assessment are a priority to prevent autistic individuals from falling through the cracks of suicide prevention efforts.

### Suicide Crisis Services

There are several potentially important points of contact for an individual in a suicidal crisis, including crisis hotlines, law enforcement services, and EDs. Given high rates of STB in autistic people, it is critical to optimize these acute-care services to best support autistic individuals in crisis.

### Suicide Hotlines and Crisis Support

Suicide prevention hotlines and crisis support services are part of the first line of defense for supporting an individual in a suicidal crisis. Despite this, Cleary et al. [54] note that across 28 studies examining how autistic individuals access mental health services, none mention accessing hotlines and only two studies briefly mention crisis support services [55, 56]. However, the authors highlight publicly available guidelines for professionals to help autistic individuals through a crisis [57]. In a survey of all police departments and youth servicing organizations in two East Coast counties, Hassrick et al. [58] found that while half of the police departments coordinated care for autistic youth in suicidal crisis, care coordination between police departments and other systems was variable. Both articles highlight the lack of literature examining what the first steps in effective crisis care look like when supporting autistic individuals.

### ED and Inpatient Care Settings

Using data from the Nationwide Emergency Department Sample (NEDS), Schott et al. [59] found that self-injury (suicidal and non-suicidal) accounted for a greater proportion of ED visits for autistic adolescents (ages 13–18) than for a random sample of same-aged youth (which included youth with and without developmental disabilities). Similarly, Cervantes, Brown, and Horwitz [38] examined NEDS data and found that ED visits coded for SI or intentional self-inflicted injury were more common among autistic youth than in a comparison group of non-autistic youth without ID. To explore the clinical presentation of autistic individuals seeking suicide-related ED services, Jachyra et al. [60] thematically analyzed 16 ED charts for autistic adults who presented with STB. Suicidal thoughts were reported for a majority of the sample (75%). One common theme was difficulties with perseveration or recurrent thoughts about STB. Interestingly, many individuals identified a social conflict or misunderstanding as the trigger for their STB, while others reported recent struggles with changes to routines and transitions. Though they noted commonalities across charts, Jachyra et al. [60] also highlighted that the nuances of each case are imperative to consider in acute care settings.

Despite high ED utilization rates in autistic individuals, there is increasing evidence that ED clinicians feel less confident providing suicide risk screening, assessment, and care to their autistic patients compared to their non-autistic patients. For example, Cervantes, Li et al. [61] found that among 16 pediatric psychiatric ED clinicians, significant feelings of low confidence for assessing STB in autistic patients were prevalent, even across clinician experience levels. The authors identified the lack of validated suicide risk assessment tools and suicide prevention approaches tailored for autistic individuals as major contributors to this issue.

Autistic individuals may also be more likely to have negative experiences in ED settings, which could contribute to heightened risk and make it more difficult for autistic individuals to collaborate with clinicians to feel safer. ED settings can be loud, crowded, and too bright, causing autistic individuals with sensory sensitivities to feel overwhelmed [54]. Some of these negative experiences could be improved by training ED staff about the varied presentations (including masking or camouflaging) and support needs of autistic individuals. Learning to decrease sensory overload, identify warning signs, and tailor information communication could improve the hospital experience for autistic individuals presenting with suicide risk [54, 57].

Autism spectrum disorder is more prevalent in adult inpatient psychiatric settings compared to the general population, as well [62]. Unfortunately, like EDs, inpatient psychiatric settings are not designed for autistic individuals’ needs, and inpatient staff are rarely trained in understanding and responding to autistic learning styles [63]. This puts autistic patients at higher risk for inappropriate care or excessive interventions, such as seclusion, restraints, unnecessary medications, and more frequent, longer admissions [63]. In contrast, specialized psychiatric inpatient programs designed to meet autistic patients’ needs may lead to shorter, less frequent inpatient stays with better clinical outcomes [63]. However, access to these programs is extremely limited, making improvements in nonspecialized inpatient settings critical. For example, routine training in understanding and responding to autistic patients’ learning styles and needs is warranted. ED clinicians should also take additional care when planning to transfer autistic patients to inpatient psychiatric facilities, including providing clear communication about what is to be expected and communicating with inpatient clinicians about how best to support each autistic patient.

### Treatment

Although evidence-based suicide prevention interventions exist, they were developed for and validated in neurotypical populations (e.g [64]). There is a significant gap in knowledge about the effectiveness of these interventions for autistic individuals [65]. While clinicians acknowledge the importance of treating STB in autistic patients, they report significantly less confidence providing suicide-related care to autistic patients compared to non-autistic patients (e.g [61]). Encouragingly, in recent years, several teams have developed tailored approaches to suicide prevention practices for autistic individuals in partnership with autistic individuals, family members, and clinicians.

#### Safety Planning

Several research groups have identified the Safety Planning Intervention [64], a widely used evidenced-based intervention, as a potentially effective suicide prevention approach for autistic individuals. For example, Schwartzman et al. [65] note that the focus on a concrete plan with outlined steps for identifying warning signs, coping strategies, and sources of support may be well-suited for autistic individuals. Safety planning also caters to various settings as it can be administered by a wide range of health professionals, typically within a single session [64]. Nonetheless, in its current form, safety planning can be viewed as too restrictive and not adaptable enough to autistic individuals’ needs. Schwartzman et al. [65] recommend several modifications including using visual aids, relying on more collaboration with caregivers, and understanding how autistic traits may lead to differences in safety plans between autistic and non-autistic individuals. There are ongoing research efforts focused on tailored approaches to safety planning that cater to autistic individuals’ strengths and preferences [66–68]. Validated modifications could contribute to increased clinician confidence in providing suicide-related care to autistic individuals and decrease ED referrals for at-risk autistic individuals.

#### Dialectical Behavior Therapy (DBT)

DBT, an evidence-based intervention that helps individuals with chronic STB, is being studied as a potentially effective treatment for autistic individuals [69]. DBT focuses on teaching and enhancing motivation to use skills across four key domains (mindfulness, distress tolerance, emotion regulation, and interpersonal effectiveness) to increase coping capacity and effective problem-solving abilities [70]. This framework aligns well with what is known about autistic individuals’ difficulties with emotion regulation, and emerging research suggests that autistic adults without ID may find DBT skills training to be feasible and acceptable [71].

In a recent study of seven autistic adults without ID who had recently engaged in self-harm and/or suicidal behaviors, the participants found DBT to be feasible and highly acceptable [72]. Of the five participants who reported SI at baseline, three reported a sustained decrease in SI frequency and one reported a decrease in SI intensity at the 4-month follow-up. In addition, no suicide attempts occurred during the study’s 8-month period. These results are encouraging for the future of treating STB in autistic people, although more research studies with larger samples are needed.

## Conclusions

While there has been increased research and improved community awareness about STB in autistic individuals, important gaps remain to fully address this life-threatening issue. We recommend that researchers now shift from focusing on STB prevalence rates to how we effectively prevent suicide in this high-risk population. An important next step is accurately screening and assessing for STB in autistic individuals. Research teams need to prioritize authentic autistic engagement and include autistic people in the development and testing of screening and assessment questions. As mentioned above, assuming that STB risk screeners and assessments designed for the general population are valid with autistic individuals may lead to false negative or false positive results. False negatives may lead clinicians to miss an opportunity to intervene and prevent a suicide, and false positives may lead autistic individuals to face unwarranted intervention (e.g., being sent to the ED, inpatient hospitalization). Both situations can lead to unintentional harm of autistic individuals (e.g., increasing STB, contributing to thwarted belongingness or hopelessness, causing unmet support needs). Ongoing efforts to test autism-specific STB risk screeners and assessments are currently underway and demonstrating promising progress. Examples include a National Institute of Mental Health (NIMH) study to develop and assess an autism-specific suicide risk screening tool [50] and an Autism Center of Excellence (ACE) study to create the first dimensional self-report questionnaire of suicidality developed for autistic adults [73].

Next, identifying protective factors is an under-researched area, but critically important to guide STB prevention and treatment efforts for autistic individuals. Centering autistic individuals’ voices and their family members will ensure that these efforts are not only prioritized, but also undertaken in a thoughtful, intentional, and affirming manner. It is particularly important to learn directly from autistic individuals with lived experience of STB and incorporate what has helped them into suicide prevention interventions and crisis supports. It is also important for research teams to collaborate with clinicians and other community partners to ensure that their studies contribute to meaningful and sustainable improvements in routine clinical care and other real-world settings. We are encouraged to see major funders (e.g., Patient-Centered Outcomes Research Institute [PCORI], NIMH) recently support this type of community-partnered research. For example, the Autistic Adults and other Stakeholders Engage Together (AASET) group is partnering with a large research team to conduct a PCORI-funded comparative effectiveness study of two tailored safety planning approaches for autistic adolescents and young adults [74], and the Academic-Autistic Spectrum Partnership in Research and Education (AASPIRE) is contributing to an NIMH-funded study to develop a community-based suicide prevention intervention for autistic individuals [75].

Finally, we recommend considering how the societal landscape may negatively impact autistic individuals’ everyday experiences through stigma, discrimination, and ableism (i.e., attitudes and practices that do not value and discriminate against people with disabilities and assume that disabled people require fixing) [76]. Relatedly, intersectional identities provide important context to understanding STB in autistic people. For example, gender identity (e.g [77, 78]) and race and ethnicity (e.g [79]) are aspects of intersectional identities that should be thoughtfully integrated into autism and STB research. Moving forward, we need a multi-faceted, comprehensive, and strengths-based approach to understanding and reducing STB in autistic people.

## Data Availability

No datasets were generated or analysed during the current study.
